# TIPARP as a prognostic biomarker and potential immunotherapeutic target in male papillary thyroid carcinoma

**DOI:** 10.1186/s12935-024-03223-6

**Published:** 2024-01-17

**Authors:** Jianlin Zhang, Xumin Zhou, Fan Yao, JiaLi Zhang, Qiang Li

**Affiliations:** General Surgery Center, Department of Thyroid Surgery, Zhujiang Hospital, Southern Medical University, 253 Gongye Middle Avenue, Haizhu District, Guangzhou, Guangdong 510280 China

**Keywords:** Papillary thyroid carcinoma, Sex disparity, Lymph node metastasis, Immunity

## Abstract

**Background:**

Male patients with papillary thyroid carcinoma (PTC) tend to have poorer prognosis compared to females, partially attributable to a higher rate of lymph node metastasis (LNM). Developing a precise predictive model for LNM occurrence in male PTC patients is imperative. While preliminary predictive models exist, there is room to improve accuracy. Further research is needed to create optimized prognostic models specific to LNM prediction in male PTC cases.

**Methods:**

We conducted a comprehensive search of publicly available microarray datasets to identify candidate genes continuously upregulated or downregulated during PTC progression in male patients only. Univariate Cox analysis and lasso regression were utilized to construct an 11-gene signature predictive of LNM. TIPARP emerged as a key candidate gene, which we validated at the protein level using immunohistochemical staining. A prognostic nomogram incorporating the signature and clinical factors was developed based on the TCGA cohort.

**Results:**

The 11-gene signature demonstrated good discriminative performance for LNM prediction in training and validation datasets. High TIPARP expression associated with advanced stage, high T stage, and presence of LNM. A prognostic nomogram integrating the signature and clinical variables reliably stratified male PTC patients into high and low recurrence risk groups.

**Conclusions:**

We identified a robust 11-gene signature and prognostic nomogram for predicting LNM occurrence in male PTC patients. We propose TIPARP as a potential contributor to inferior outcomes in males, warranting further exploration as a prognostic biomarker and immunotherapeutic target. Our study provides insights into the molecular basis for gender disparities in PTC.

**Supplementary Information:**

The online version contains supplementary material available at 10.1186/s12935-024-03223-6.

## Background

Thyroid cancer, with rising incidence globally, is the most prevalent malignancy of the endocrine system [1]. In China, thyroid cancer ranked as the seventh most common cancer in 2020, with 221,000 new cases as per the Global Cancer Statistics report [2]. Differentiated thyroid cancers (DTC) comprise over 95% of thyroid carcinomas, with papillary thyroid carcinoma (PTC) being the predominant histological subtype [3]. Currently, surgery is the primary treatment for PTC, either total or near-total thyroidectomy, or unilateral lobectomy with isthmectomy, based on tumor extent, patient age and comorbidities [4].

Despite an overall favorable prognosis, PTC presents risks such as lymph node metastasis (LNM) that impair quality of life and prognosis [5]. Conventional diagnostic approaches for LNM like clinical exam, ultrasound, and CT have limited accuracy, necessitating invasive fine needle aspiration (FNA) confirmation [6]. However, FNA has high technical demands and sample quality requirements. Additionally, consensus is lacking on prophylactic lymph node dissection during surgery, especially for preoperative lymph node-negative patients. Numerous studies demonstrate increased risks of recurrent laryngeal nerve/parathyroid damage, sometimes permanent hypocalcemia and vocal cord paralysis, with lymph node clearance [7]. Thus, preoperative LNM risk assessment is critical for guiding surgical protocols and prognosis in PTC. With advancing medical technology, several studies show combining FNA and genetic testing can effectively improve diagnostic accuracy [8].

There is a pronounced gender disparity in thyroid cancer incidence, with significantly higher rates in females versus males [9]. However, recent evidence indicates male thyroid cancer patients experience greater invasiveness and poorer prognosis [10], though the mechanisms underlying this difference remain unclear [11]. A Chinese retrospective study identified male sex as an independent risk factor for central cervical lymph node metastasis in papillary thyroid carcinoma [12]. Large cohort studies also associate male gender with higher rates of revision neck surgery for differentiated thyroid cancers [13]. Extensive research has focused on elucidating the heightened female papillary thyroid cancer (PTC) incidence, yet few studies have delineated the inferior male prognosis [14]. One study by Wang et al. leveraged bioinformatics to uncover significant expression differences between male and female PTC patients. Additionally, some differentially expressed viral response genes also demonstrated gender-specific prognostic value in PTC [15].

In this study, we integrated seven GEO datasets and the TCGA-THCA cohort to identify reliable differentially expressed genes (DEGs) specific to male PTC patients, termed msDEGs. Furthermore, we performed univariate Cox regression and lasso logistic regression analysis to pinpoint msDEGs associated with LNM in PTC. Utilizing gene expression and clinical data from TCGA-THCA, we then developed a gene signature-based predictive model for LNM. We also established TIPARP as a candidate LNM-related gene, validated by immunohistochemistry. Additional gene set enrichment analysis (GSEA) elucidated biological functions and pathways linked to TIPARP. Finally, we assessed correlations between TIPARP expression, immune infiltration, and the tumor microenvironment.

## Method

### Data collection

We systematically searched the Gene Expression Omnibus (GEO) database (http://www.ncbi.nlm.nih.gov/geo/) from inception to September 1, 2022 to identify relevant gene expression profiles. Datasets were included if they met the following criteria:

1. Contained data for both normal thyroid and tumor samples.
2. Included both female and male samples.
3. Used Affymetrix gene chips for detection.
4. Had sample sizes greater than 10.

Based on these criteria, we screened and selected seven GEO datasets: GSE3467, GSE3678, GSE6004, GSE29265, GSE33630, GSE53157, and GSE60542. Additionally, an RNA-sequencing dataset from The Cancer Genome Atlas (TCGA) (https://cancergenome.nih.gov/) was chosen as an independent validation cohort [16].

### Data processing

The gene expression profiles from GEO datasets were downloaded and normalized using R software (version 4.2.1). Clinical data and survival information for the TCGA-THCA cohort were obtained from UCSC Xena (https://xena.ucsc.edu; University of California, Santa Cruz). The clinical data included age at diagnosis, sex, number of examined lymph nodes, tumor focus, thyroid tumor location, pathologic TNM staging (AJCC 7th edition), and progression-free interval (PFI) [17]. Probe IDs were matched to gene symbols using GEO platform annotations (ftp://ftp.ncbi.nlm.nih.gov/geo/platforms). For probes mapping to the same gene, expression values were averaged. For samples with missing gender information, the massiR Bioconductor package was utilized to predict sex based on unsupervised clustering of Y chromosome probe signals [15].

### Differentially expressed genes (DEGs) screening and funtional annotation

The R package “limma” was utilized to identify differentially expressed mRNAs between normal and PTC samples in females and males separately. Cutoffs of false discovery rate (FDR) < 0.05 and |log2Fold change| >1 were applied. Genes significantly differentially expressed between PTC and normal tissues exclusively in males were selected as male-specific DEGs (msDEGs). Results from the 7 GEO datasets were merged and intersected with msDEGs from TCGA-THCA to determine common msDEGs.

The Bioconductor package “clusterProfiler” annotated msDEG biological functions via gene ontology (GO) and Kyoto Encyclopedia of Genes and Genomes (KEGG) pathway enrichment analysis, exploring the terms biological process (BP), cellular component (CC), and molecular function (MF) [18]. An FDR < 0.05 was considered significant [18].

### Signature construction and validation

Following differential expression analysis, univariate logistic regression screened for genes significantly associated with LNM (*P* < 0.05). The 124 male PTC cases from TCGA-THCA were then randomly partitioned into training (70%) and testing (30%) sets. Least absolute shrinkage and selection operator (LASSO) regression selected crucial LNM-related genes and coefficients from the msDEGs [19], while optimal λ was choosen using cross-validation by minimum(lambda.min) and 1-SE- criteria (lambda.1se). A novel gene signature was derived via multivariate logistic regression, with risk scores calculated using the gene coefficients in R package “rms”.

Signature predictive performance was evaluated by area under the receiver operating characteristic curve (AUC) in the training, testing, and additional validation set (GSE60542). Calibration curves assessed prediction accuracy.

### Comprehensive analysis of the model

We analyzed correlations between the gene signature and clinical parameters including age, gender, BRAF/RAS mutation status, TNM stage, extrathyroidal extension, residual tumor, and primary tumor foci in the TCGA-THCA cohort [20]. Patients were classified as low or high recurrence risk via optimum risk score cutoffs from R package “qROC”. Single sample gene set enrichment analysis (ssGSEA) with R package “gsva” calculated immune cell type and pathway infiltration scores [21]. Gene set enrichment analysis (GSEA) was then performed between high and low risk groups using GSEA v4.1 software against the KEGG reference gene sets [22]. Significantly enriched pathways were defined as nominal *p*-value < 0.05 and FDR < 0.05.

### Building and validation of a predictive nomogram

To identify potential clinical and genomic indicators of lymph node metastasis (LNM) in papillary thyroid carcinoma (PTC), multivariate logistic regression was performed incorporating clinical variables and the gene signature-derived risk score using R package “Stats”. A nomogram to predict LNM probability was constructed using R package “rms” (v6.1-0) [23]. Calibration curves were plotted to assess nomogram prediction performance. The Hosmer-Lemeshow goodness-of-fit test (R package “ResourceSelection”) evaluated calibration and prediction accuracy.

### IHC staining and evaluation

We also examined TIPARP protein levels through immunohistochemistry (IHC) in 40 paired papillary thyroid carcinoma (PTC) and normal thyroid tissues from Zhujiang Hospital collected from February 2021 to March 2023 after ethical approval and informed consent.

Eighty human thyroid normal and tumor tissues were fixed in 4% formaldehyde and embedded in paraffin. Paraffin-embedded samples were cut into 6 μm thick sections, and then they were placed in xylene and ethanol solution for deparaffinating and rehydration in PBS. 3% hydrogen peroxide solution were used to block endogenous peroxidase activity and nonspecific sites after samples were placed in the EnVisionTM FLEX Target Retrieval Solution, High pH(50×) for antigen retrieval [24].

Sections were stained with rabbit antibodies against TIPARP (Abcam, AB84664, 1:500), for incubating at 4 °C overnight, and then incubation in the second antibody was carried out at room temperature for 2 h. DAB solution was used to develop and hematoxylin was re-stained.

Results were visualized and recorded with the help of digital pathology section scanner(d.metrix DMS-10-Pro). Ten pictures of thyroid normal or tumor tissues foci were randomly taken for each slide and images were analyzed using digital image analyzing software (ImageJ, U.S. National Institute of Health, Bethesda, Maryland, USA, https://imagej.net) [25], and the mean option density was calculated in the manner of integrated option density (IOD) [26].

### Immune microenvironment and gene set enrichment analysis

To assess lymph node metastasis (LNM) risk stratification by TIPARP, papillary thyroid carcinoma (PTC) cases were classified as “low risk” or “high risk” based on median TIPARP expression. Single sample gene set enrichment analysis (ssGSEA) with R package gsva then calculated infiltration scores for 16 immune cell types and 13 immune-related pathways [21, 27].

Additionally, gene set enrichment analysis (GSEA) was performed between high and low TIPARP expression groups using GSEA v4.1 against the KEGG reference gene sets [28]. Significantly enriched pathways were defined as nominal *p*-value < 0.05 and false discovery rate (FDR) < 0.05 [29].

### Single-cell data analysis

We searched the GEO database for single-cell RNA-sequencing datasets from male papillary thyroid carcinoma (PTC) patients with lymph node metastasis (LNM) and obtained raw data for GSE193581, GSE163203, and GSE191288 [30–32]. The R package harmony was used to integrate the data from each sample. After dimensionality reduction, clustering, and cell type annotation with “SingleR” and CellMarker2.0 database(http://bio-bigdata.hrbmu.edu.cn/CellMarker/) [33], we assigned each cell population to specific cell types. Violin plots generated with “Seurat” visualized gene expression across annotated cell types [34, 35].

### Chemotherapy and immunotherapy sensitivity analysis

To investigate TIPARP-associated drug sensitivity in male papillary thyroid carcinoma (PTC), we obtained NCI-60 cell line drug activity and RNA-seq data from CellMiner (https://discover.nci.nih.gov/cellminer) [36]. Pearson correlation analysis examined sensitivity for FDA-approved or clinical trial drugs [37].

We also calculated tumor mutational burden (TMB) in TCGA-THCA using TCGAbiolinks and correlated it with TIPARP expression [38]. The Estimation of System Immune Response (EaSIeR) method predicted immunotherapy response based on the tumor microenvironment to assess associations with TIPARP expression [38, 39].

### Statistical analysis

All statistical analyses were performed in R v4.2.1 (https://www.r-project.org/). Differentially expressed genes (DEGs) were identified using the limma package (v3.6). LASSO regression selected candidate genes predicting lymph node metastasis (LNM). After developing the gene signature model, multivariate logistic regression assessed the value of the risk score and clinical variables for predicting LNM. Receiver operating characteristic (ROC) curves were generated with pROC (v1.17.0.1) to evaluate model accuracy, with the optimum sensitivity + specificity threshold identified. Comparisons between two and among three or more groups were conducted using the two-tailed Student’s t-test. *P* < 0.05 was considered statistically significant.

## Result

### 1.356 male specific differentially expressed genes

We analyzed 7 GEO datasets and TCGA-THCA, using GSE60542 for validation. Of 7 datasets, 245 samples lacked sex annotation and were predicted (massiR package), giving 365 female and 159 male samples (Supplementary Table 1). A stepwise approach identified male-specific differentially expressed genes (msDEGs) associated with lymph node metastasis (LNM) in papillary thyroid carcinoma (PTC).

**Fig. 1:**
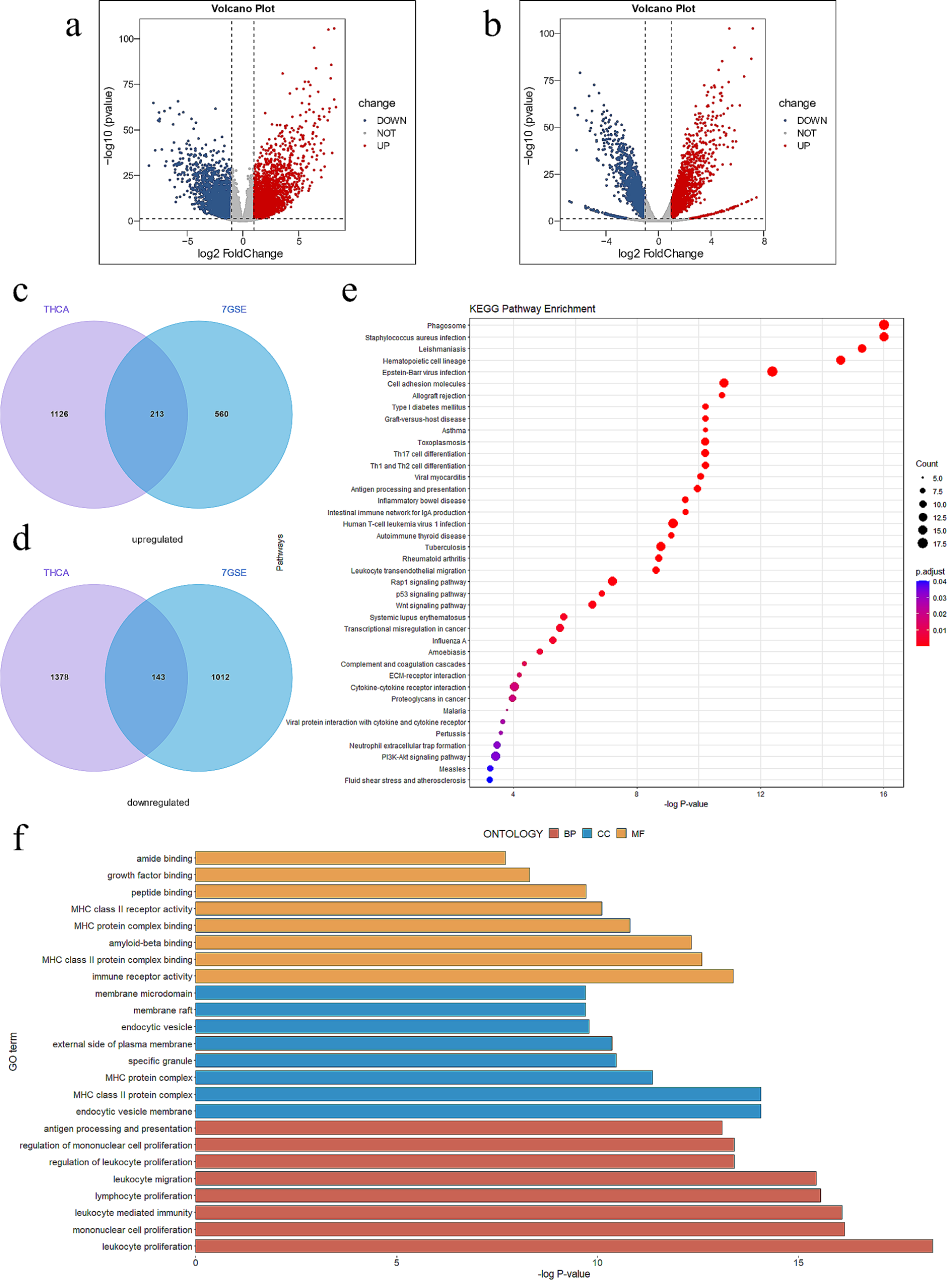
Identification of the msDEGs in TC and data integration. (**a**) Volcano plot of DEGs in male in THCA, (**b**) Volcano plot of DEGs in female in THCA, (**c**) Venn plots of overlapping upregulated msDEGs, (**d**) Venn plots of overlapping downregulated msDEGs, (**e**) Bubble charts of the enriched KEGG pathways. (**f**) Bar plot of the enriched GO terms

In the 7 GEO datasets, 1860 msDEGs were found between male tumor and normal tissue. TCGA-THCA yielded 2890 msDEGs from 5856 DEGs in male and 3342 in female (Fig. 1a,b). By intersecting GEO and TCGA results, 383 total msDEGs (213 upregulated, 143 downregulated) remained (Fig. 1c,d). GO and KEGG pathway enrichment analysis annotated the 383 msDEGs (Fig. 1e-f). Top GO terms included immune receptor activity, MHC protein binding, leukocyte proliferation and migration. Key enriched pathways were PI3K-Akt signaling, microRNAs in cancer, and proteoglycans in cancer.

### Construction of the predictive model

**Fig. 2:**
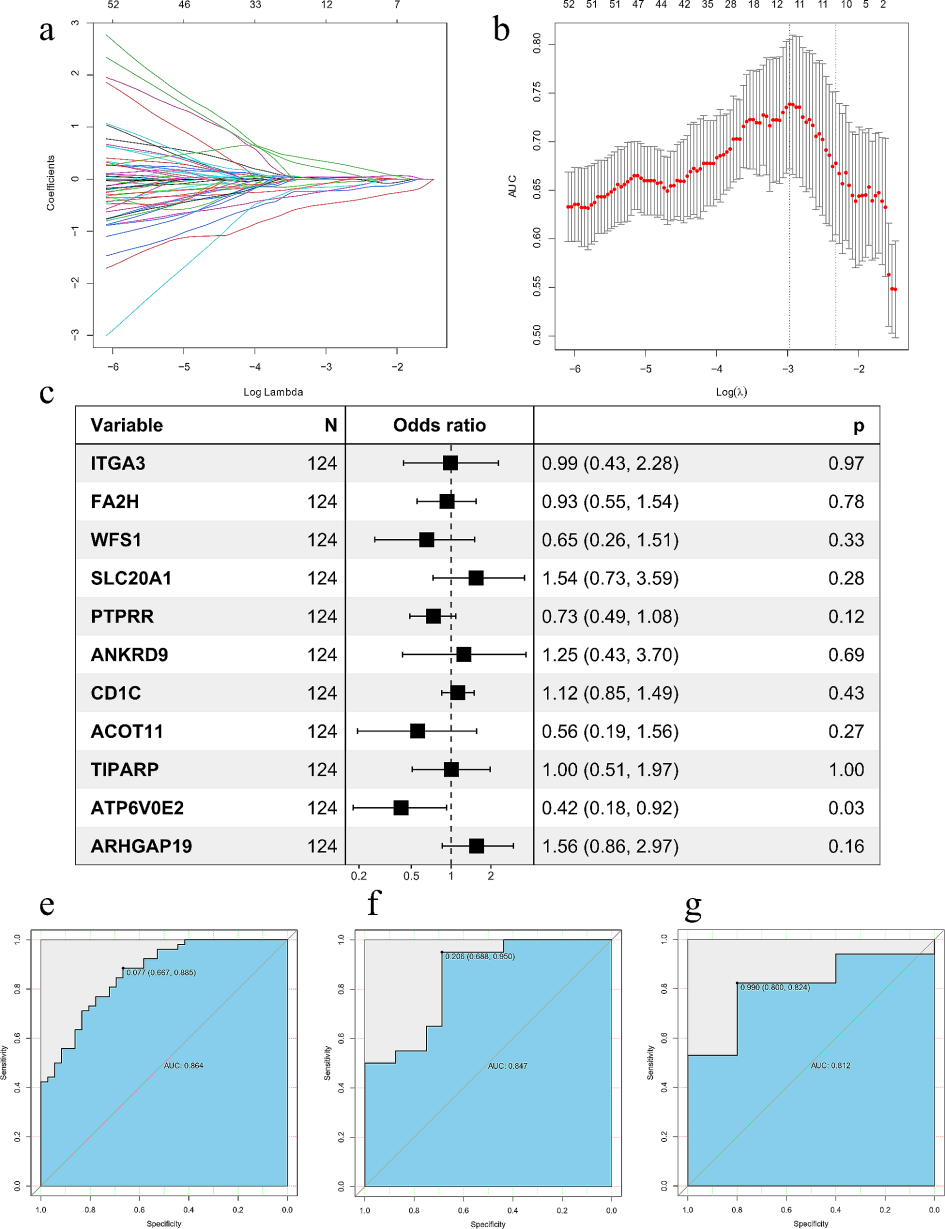
Establishment of a 11-gene model for predicting the LNM of male PTC patients. (**a**) LASSO coefficient profiles of the 209 msDEGs. (**b**) Selection of the optimal λ-value through the cross-validation. The best values by minimum(lambda.min, right vertical dotted line) and 1-SE- criteria (lambda.1se, left vertical dotted line) representing the dotted vertical lines. (**c**) the forest plot showing the results of the univariate Logistic regression analyses. (**e**~**g**) The ROC of the training group (AUC = 0.864); The ROC of the testing group (AUC = 0.847); The ROC of the validation group (AUC = 0.812).

Using Univariate logistic regressionthe, 209 of 383 msDEGs are closely related to lymph node metastasis (LNM)(*p* < 0.05). The dataset with survival data was randomly split into training (70%) and test (30%) sets. msDEGs associated with LNM underwent LASSO regression to reduce overfitting and feature dimensions (Fig. 2a,b). By preferring to lambda.1se considering the best AUC values in lasso model, we developed an 11-gene signature model to predict LNM in male PTC (Fig. 2c). Risk scores were calculated based on the signature.

Receiver operating characteristic (ROC) curves assessed signature performance in predicting LNM. Area under the curve (AUC) was 0.864, 0.847 and 0.855 for training, test and combined sets, respectively (Fig. 2E-G). Performance was verified in the validation set (AUC 0.812).

### Comprehensive analysis of the 11 gene model

Analyzing signature correlations with clinical variables showed lower risk scores in patients without extrathyroidal invasion versus those with invasion (*p* < 0.001). Scores were similar across groups stratified by age (*p* = 0.116), BRAF mutation (*p* = 0.276), and RAS mutation (*p* = 0.656).

**Fig. 3:**
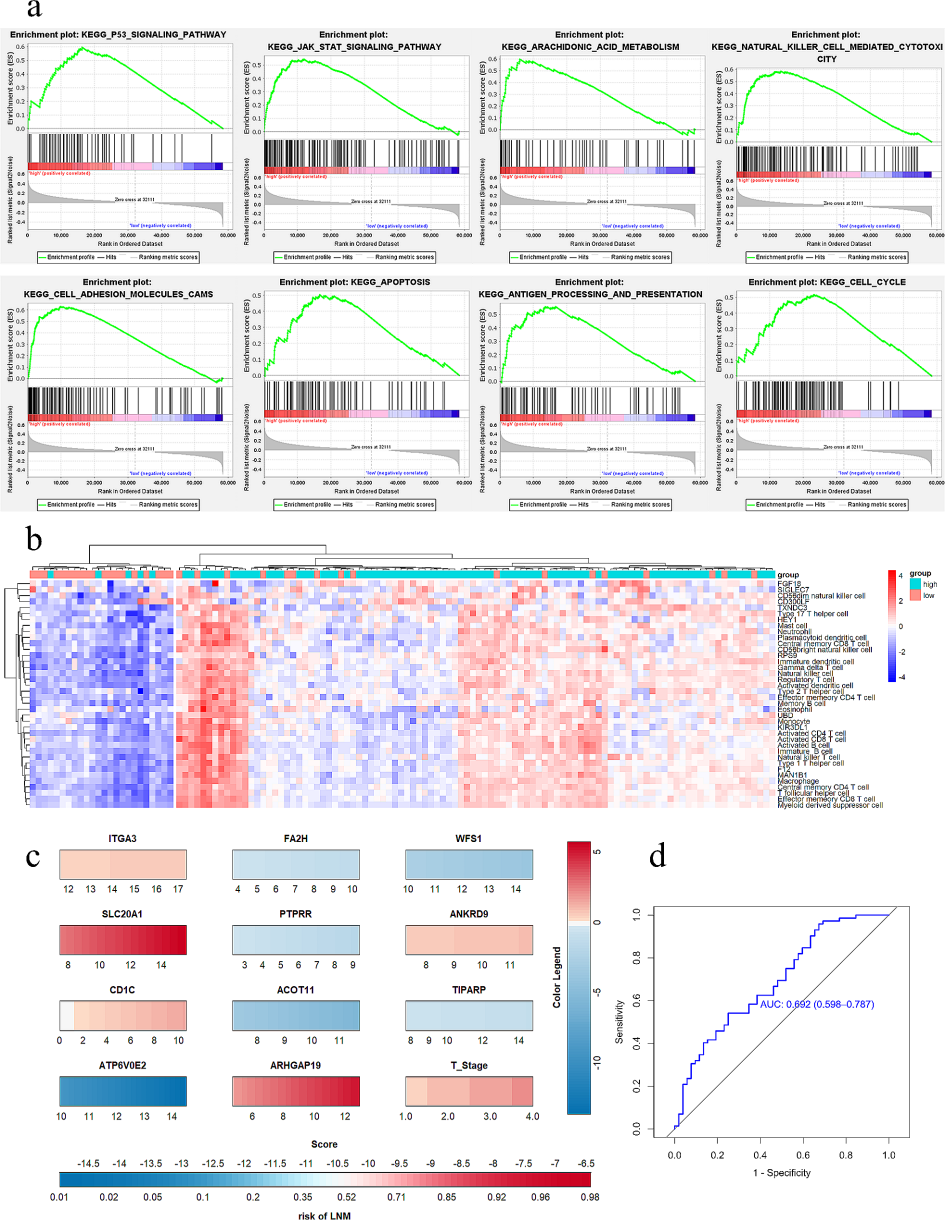
Exploration of the 11-gene model. (**a**) GSEA revealed the signaling pathways enriched in the high-risk group. (**b**) Comparison of the ssGSEA score of signatures in the high- and low-risk score patients in TCGA cohorts. (**c**) nomogram used for prediction of LNM in male PTC patients. (**d**) The ROC of the TIPARP (AUC = 0.692)

Gene set enrichment analysis (GSEA) of 124 TCGA-THCA male cases revealed altered pathways in the high-risk group. Enrichment of p53 signaling (NES 1.90, *p* < 0.001) and JAK-STAT signaling (NES 1.81, *p* = 0.008) suggests aggressive behavior (Fig. 3a, Additional file 1). This implicates potential mechanisms for the 11-gene signature in male PTC.

Divergence between risk groups likely stems from tumor microenvironment (TME) intricacies. Single sample GSEA (ssGSEA) examined infiltration of 28 immune cell types in TCGA thyroid carcinomas. The high-risk group showed significantly higher immune infiltration, indicated by elevated immune cell activation (Fig. 3b).

### Nomogram

To enable clinical application, a nomogram integrating the 11-gene signature and clinical-pathological risk factors (gender, age, stage, T, N, M) predictive of lymph node metastasis (LNM) by univariate logistic regression was developed.

Nomogram predictive performance was evaluated through 1000 bootstrap resampled calibration curves, showing good agreement between predicted and observed LNM. The Hosmer-Lemeshow test indicated good calibration (*p* = 0.319). The nomogram c-index was 0.786. Risk scores were calculated per sample and receiver operating characteristic (ROC) curves generated (Fig. 3c).

### Analysis and validation of TIPARP expression in PTC patients

Gene function analysis revealed TIPARP’s links to gender differences, androgen and estrogen activity. TCGA data analyzed through XENA tools showed TIPARP mRNA was upregulated in male papillary thyroid carcinoma (PTC) with lymph node metastasis (LNM) versus without (AUC 0.692), indicating potential as a diagnostic biomarker (Fig. 3D). High TIPARP associated with male sex, advanced stage, high T stage, and more LNM.

**Fig. 4:**
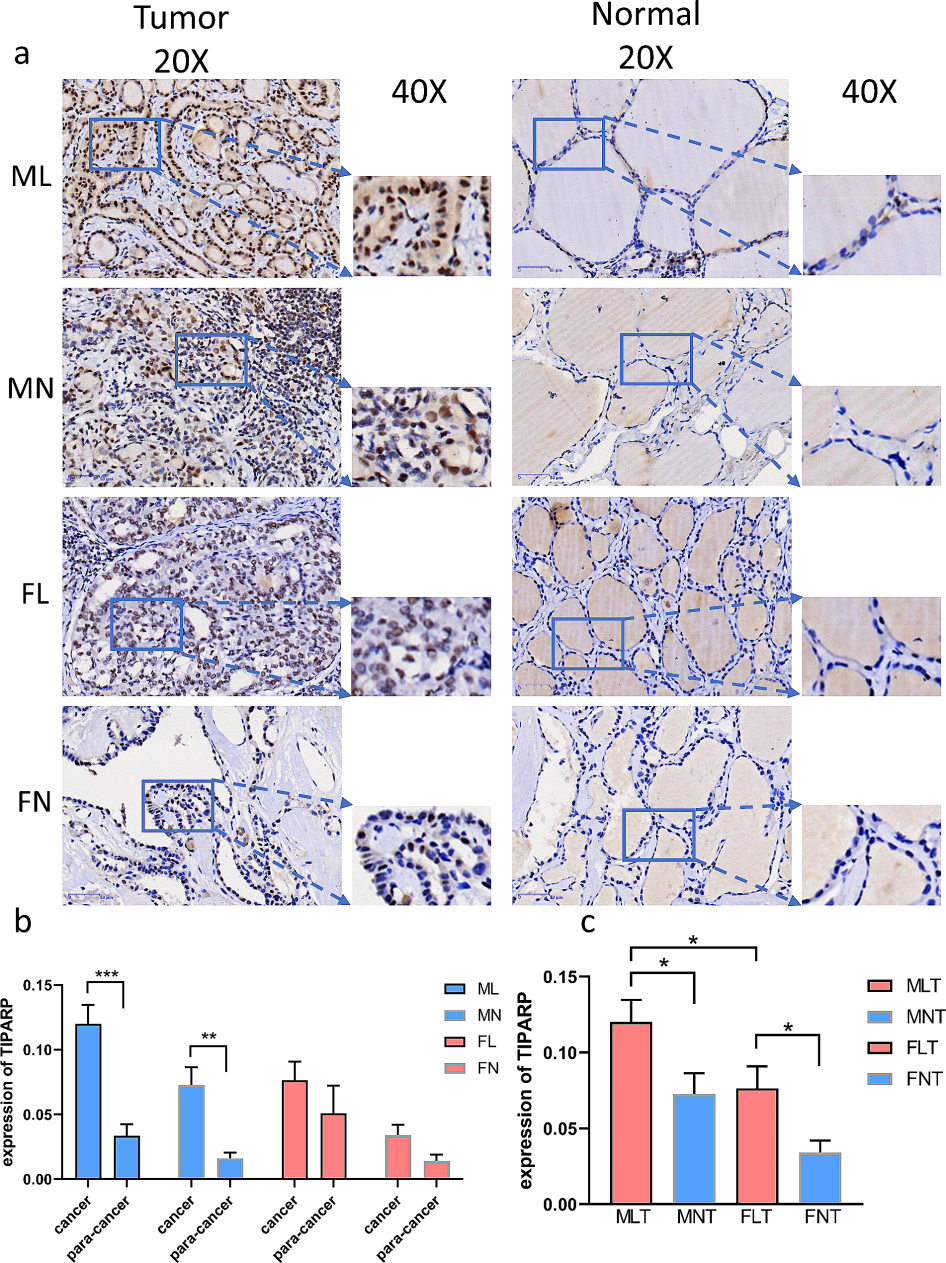
Verification the expression of TIPARP. (**a**-**b**) Representative sections of PTC thyroid, normal tissues. The expression of TIPARP was detected by using immunohistochemistry (IHC). (**c**) compare of TIPARP in different tumor group. (*P < 0.05; **P < 0.01, ***P < 0.001) (ML: Male lymph node metastasis group; MN: Male non-lymph node metastasis group; FL: Female lymph node metastasis group; FN: Female non-lymph node metastasis group)

Immunohistochemistry verified increased TIPARP protein expression in human PTC versus adjacent normal tissue, especially in males (Fig. 4A-C, E). Notably, TIPARP expression was substantially elevated in male PTC patients with LNM compared to those without (Fig. 4F).

### Correlation of TIPARP expression with tumor immune microenvironment

Emerging research underscores the pivotal role of the tumor microenvironment in immunotherapy response. The tumor microenvironment also impacts tumor migration. To further understand links between TIPARP expression and immune activity, we calculated enrichment scores for immune cell subsets and immune-related functions/pathways by ssGSEA algorithm.

**Fig. 5:**
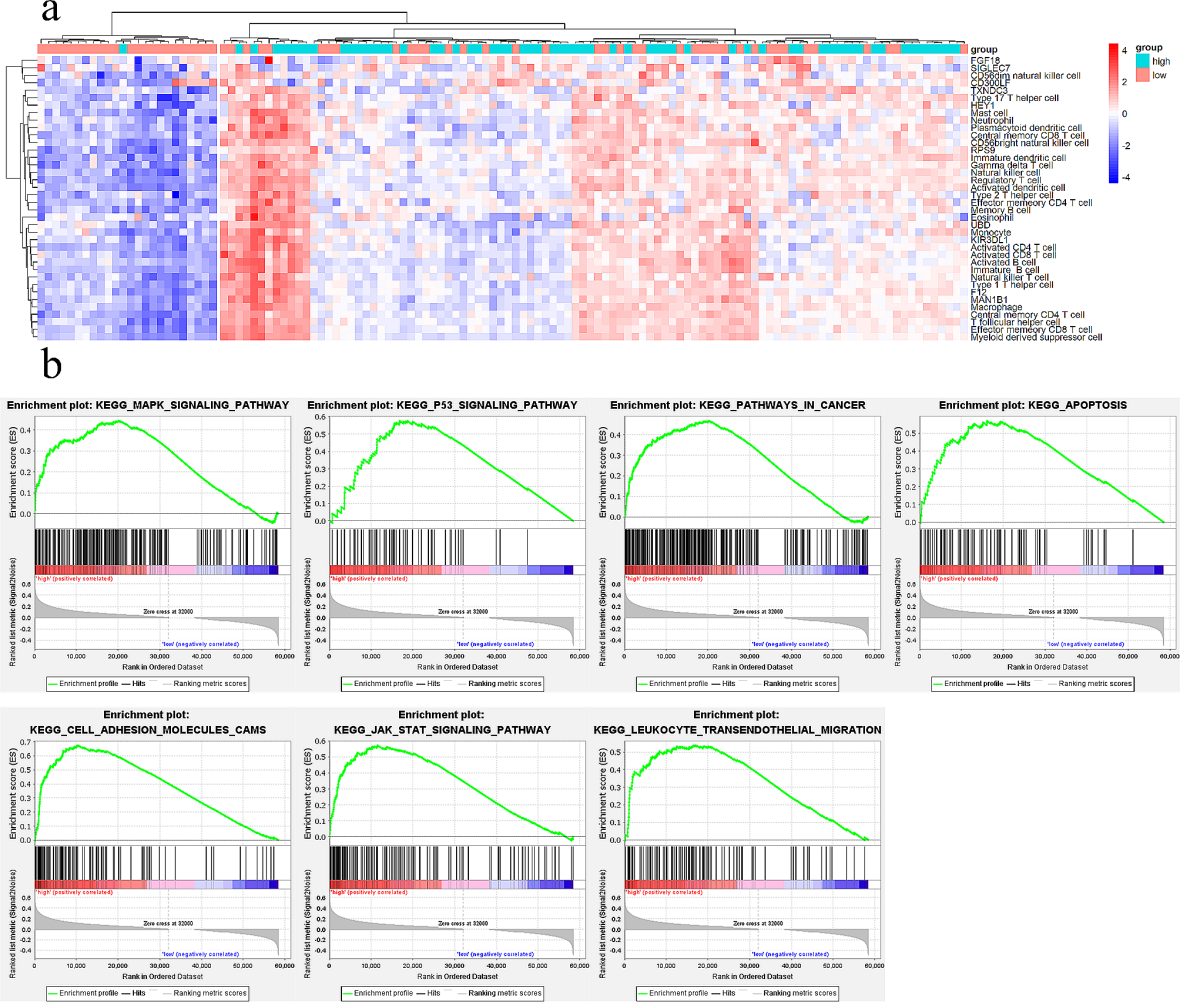
ssGSEA and GSEA scores between the different TIPARP expression groups. (**a**) Comparison of the ssGSEA score of signatures in the high- and low- TIPARP expression patients in TCGA cohorts. (**b**) GSEA showed that high TIPARP expression was positively correlated with several immunity-related and cancerrelated pathways

Intriguingly, the high TIPARP expression group showed positive correlations with immune cell infiltration. The ssGSEA results further confirmed the signature’s reflection of tumor immune microenvironment status (Fig. 5A).

### Biological function of TIPARP in male PTC

To identify pathways potentially regulated by TIPARP, we performed gene set enrichment analysis (GSEA) comparing tissues with high versus low TIPARP expression. Using thresholds of normalized enrichment score (NES) > 0 and nominal *p*-value < 0.05, enriched pathways were identified.

High TIPARP expression associated with cell cycle, TGF-beta signaling, ErbB signaling, RIG-I-like receptor signaling, and p53 signaling (Fig. 5B, Additional file 2).

### Single-cell data analysis

**Fig. 6:**
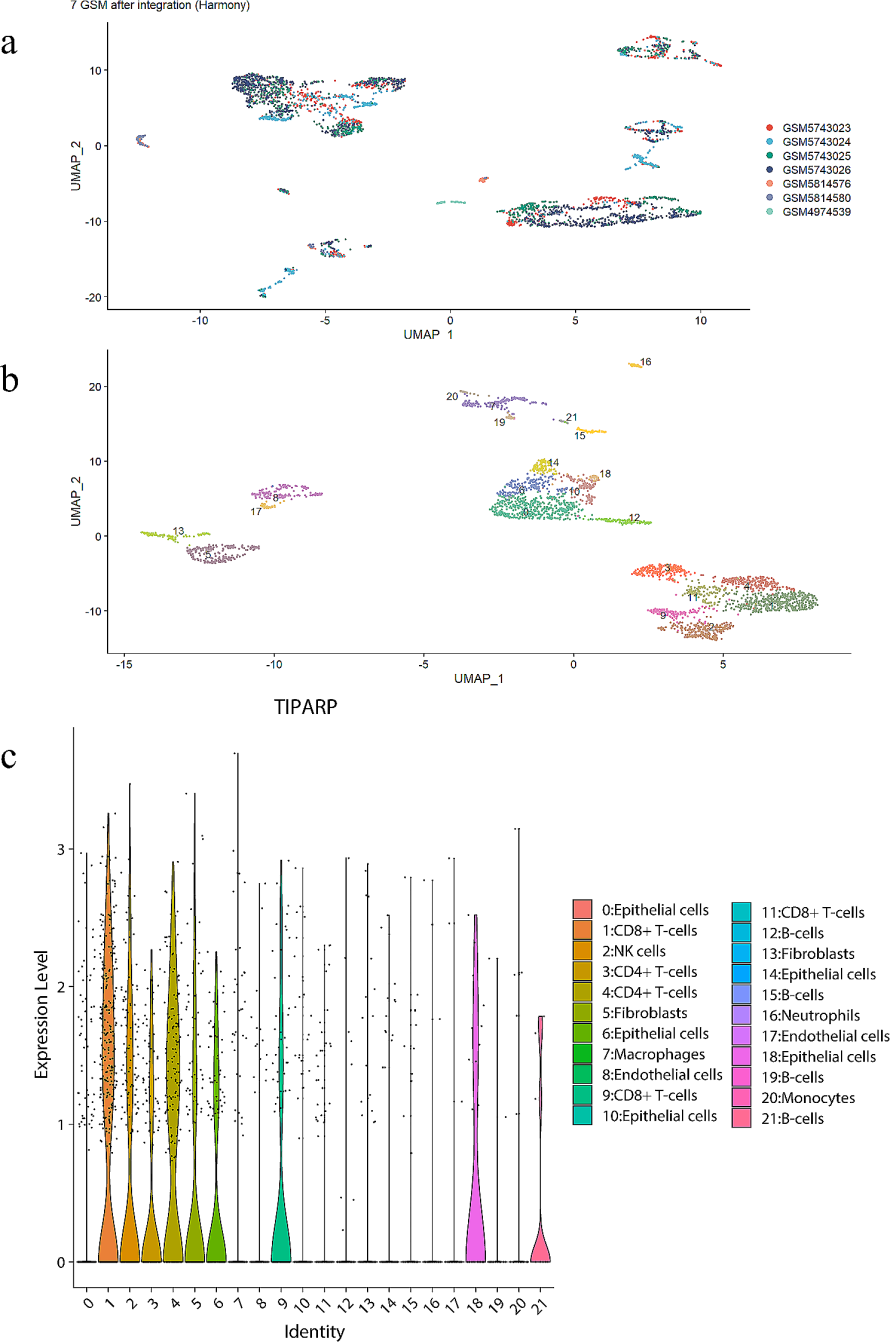
single-cell analysis. (**a**) Scatter plot showed the result of the combination of 7 GSM. (**b**) Scatter plot showed the distributions of different cell types of the combined dataset. (**c**) Violin plots of TIPARP expression in various types of annotated cells in the combined datasets

We obtained raw single-cell RNA-seq data for 7 male papillary thyroid carcinoma samples with lymph node metastasis from GEO datasets GSE193581, GSE163203, and GSE191288. After series clustering and dimensionality reduction, 22 total cell clusters were identified (Fig. 6A). Using SingleR and CellMarker2.0 database, clusters were annotated to various cell types (Fig. 6B).

In the merged data from 7 GSM in GEO dataset, TIPARP was expressed across multiple annotated immune and epithelial cell populations (Fig. 6C), partially validating associations between TIPARP and immune activity in male PTC.

### Drug sensitivity analysis

**Fig. 7:**
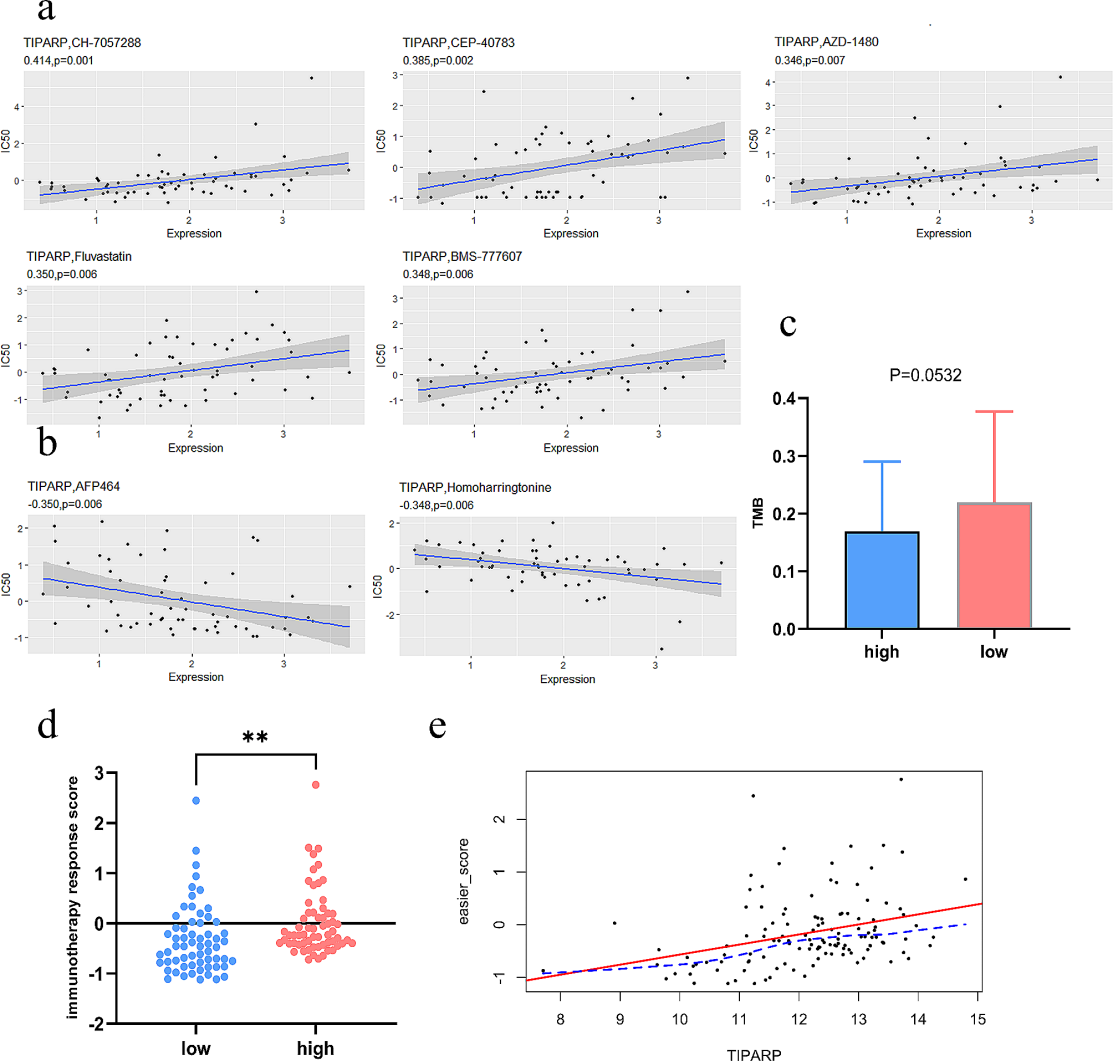
Drug and immunotherapy response analysis. (**a**-**b**) The correlation between drug sensitivity and TIPARP in Cellminer database. (**c**) Correlation of the TIPARP expression with tumor mutation burden (TMB) level in the TCGA cohort. (**d**) Correlations of the easier score with TIPARP expression. (**e**) Scatter plot of TIPARP vs. easier score

We analyzed correlations between TIPARP expression and drug sensitivity using the CellMiner database. TIPARP expression positively correlated with CH-7,057,288, CEP-40,783, Fluvastatin, BMS-77,760, and AZD-1480 (Fig. 7A), but negatively correlated with AFP464 and Homoharringtonine (Fig. 7B).

The data suggest TIPARP may confer chemoresistance to agents like Fluvastatin, while reduce chemoresistance to antitumor drugs like Homoharringtonine, and we speculate that the influence of TIPARP on chemoresistance might be linked to biosynthesis of DNA and RNA, and lipid metabolism.

### Associations between TIPARP and the efficacy of immunotherapy

Tumor mutational burden (TMB) serves as a biomarker for immunotherapy response in some cancers, but TIPARP did not correlate with TMB (Fig. 7C). Using the R package EaSIeR, we objectively evaluated predicted immunotherapy response by integrating multiple immune proxies including cancer type, TMB, pathway activities, cell fractions, transcription factor activities, and intra/intercellular signaling.

Notably, high versus low-risk groups stratified by TIPARP expression showed significant differences in EaSIeR scores (Fig. 7D). TIPARP expression positively associated with EaSIeR scores (Fig. 7E).

## Discussion

Papillary thyroid carcinoma (PTC) demonstrates distinct gender differences, with higher incidence in females but poorer outcomes in males. The reasons underlying this disparity remain unclear. Lymph node metastasis (LNM), a major manifestation of PTC progression, also shows higher rates in males. The lack of definitive LNM diagnosis poses challenges for surgical management. While numerous studies have examined LNM prediction in general PTC populations, few have explored the mechanisms driving increased LNM in male PTC specifically. In this study, through comprehensive analysis of public PTC datasets, we identified a set of genes differentially expressed only in males that associate with LNM occurrence.

Recent advances in bioinformatics have enabled discovery of novel biomarkers for papillary thyroid carcinoma (PTC) through extensive genomic profiling. Our integrated analysis of GEO and TCGA datasets identified 356 robust differentially expressed genes (DEGs) implicated in PTC pathogenesis. KEGG pathway analysis revealed enrichment for tumorigenesis pathways.

Based on univariate and multivariate Cox regression, an 11-gene signature model stratified male PTC patients into high and low-risk groups for lymph node metastasis (LNM), with significant prognostic performance by ROC analysis. Compared to a previous 14-gene signature, our model demonstrated improved predictive accuracy (c-index 0.855 vs. 0.806), highlighting utility as a reliable LNM predictor in male PTC.

The signature also associated with important clinical factors like extrathyroidal invasion and TNM stage. Tumor microenvironment analysis revealed greater immune cell infiltration in the high-risk group, including T cells, NK cells, macrophages, eosinophils, mast cells, MDSCs and dendritic cells.

GSEA showed enrichment for cell adhesion, NK cytotoxicity, JAK/STAT, apoptosis and p53 signaling in the high-risk group, linking the 11 genes to tumor progression. A prognostic nomogram integrating the signature and T stage was developed for individualized LNM risk estimation.

Functional analysis identified TIPARP as a key candidate gene warranting further exploration. TIPARP encodes a poly(ADP-ribose) polymerase regulating innate immunity and repressing type I interferon signaling [40]. It also modulates stem cell pluripotency, autophagy and gene expression, with induction by growth factors, viral infection, nuclear receptors, hypoxia and aryl hydrocarbon receptor (AHR) [41]. Prior studies establish TIPARP as a negative regulator of AHR and IFN-I signaling [42, 43].

GO and KEGG enrichment linked TIPARP to cellular hormone metabolism including androgen and estrogen. Immunohistochemistry validated TIPARP protein upregulation in male versus para-carcinoma thyroid tissues.

Additional GSEA analysis revealed TIPARP’s potential impacts on apoptosis, JAK/STAT, p53, cell adhesion, MAPK, and immune cell migration pathways. Literature indicates TIPARP can suppress Warburg effect and tumorigenesis by inhibiting HIF-1 [44], exhibits tight regulation by AR signaling in prostate cancer [45, 46], and correlates with antitumor immunity .

Single-cell RNA sequencing of 7 male lymph node metastatic PTCs showed TIPARP upregulation primarily in malignant, T cell and cholangiocyte populations [47]. This implicates TIPARP’s involvement in PTC progression through interactions with these cell types, warranting further exploration through functional experiments.

Radioactive iodine is first-line for metastatic papillary thyroid cancer, but 60% develop resistance necessitating new strategies [48]. In some models, TIPARP inhibitors show persistent tumor growth suppression, potent antiproliferative effects, and restored interferon signaling [49]. Our CellMiner analysis revealed correlations between TIPARP and drug sensitivities like Homoharringtonine and Fluvastatin.

Immunotherapy also shows promise for advanced thyroid cancers, underscoring the need for response biomarkers [50]. Emerging evidence proposes TIPARP activation as an anti-cancer approach, with its inhibition stimulating cancer cell and immune effects via enhanced IFN signaling [51]. Using the EaSIeR package accounting for intrinsic/extrinsic immune escape, high-risk patients stratified by TIPARP had significantly higher predicted immunotherapy efficacy scores versus low-risk.

## Conclusions

In summary, we developed and validated a prognostic 11-gene signature and diagnostic nomogram to reliably predict lymph node metastasis in male papillary thyroid carcinoma patients. Through integrated bioinformatic analysis and experimental validation, we identified TIPARP as a candidate contributor to the more aggressive phenotype in males. TIPARP’s associations with immune activity revealed by ssGSEA and single-cell analysis provide clues to understanding mechanisms underlying this disparity.

Our study lays the groundwork for elucidating biological drivers of inferior outcomes in male PTC patients. The gene signature and nomogram may enable better risk stratification to guide surgical management. TIPARP warrants further exploration as both a prognostic biomarker and potential immunotherapeutic target for this understudied subgroup.

Overall, our findings offer insights into the molecular underpinnings of gender disparities in PTC. With further research, this approach may inform more personalized prognostic and therapeutic strategies to improve outcomes for male patients.

Figure titles and legends.

### Electronic supplementary material

Below is the link to the electronic supplementary material.

**Supplementary Table 1:** Table of sex distribution. The sex distribution of each GEO dataset was identified

**Additional File 1:** The result of model GSEA analysis, which contains the accurate information of the NES, p-values and FDR

**Additional File 2:**: The result of TIPARP GSEA analysis, which contains the accurate information of the NES, p-values and FDR

## Data Availability

The datasets analysed during the current study are available in the GEO database and TCGA database at [https://www.ncbi.nlm.nih.gov/geo/, https://xenabrowser.net/datapages/].
